# Predictors of Anaemia Among Young Children Receiving Daily Micronutrient Powders (MNPs) for 24 Weeks in Bangladesh: A Secondary Analysis of the Zinc in Powders Trial

**DOI:** 10.1111/mcn.13806

**Published:** 2025-02-10

**Authors:** Lauren Thompson, Charles Arnold, Janet Peerson, Julie M. Long, Jamie L. E. Westcott, M. Munirul Islam, Robert E. Black, Nancy F. Krebs, Christine M. McDonald

**Affiliations:** ^1^ Department of Nutrition, Graduate Group in Nutritional Biology University of California Davis California USA; ^2^ Department of Nutrition, Institute for Global Nutrition University of California Davis California USA; ^3^ Department of Pediatrics, Section of Nutrition University of Colorado Anschutz Medical Campus Aurora Colorado USA; ^4^ Nutrition Research Division International Centre for Diarrhoeal Disease Research, Bangladesh (icddr,b) Dhaka Dhaka Bangladesh; ^5^ Department of International Health, Bloomberg School of Public Health Johns Hopkins University Baltimore Maryland USA; ^6^ International Zinc Nutrition Consultative Group San Francisco California USA; ^7^ Department of Pediatrics, Division of Gastroenterology, Hepatology and Nutrition University of California San Francisco California USA

**Keywords:** anaemia, Bangladesh, micronutrient interventions, micronutrient powders

## Abstract

In Bangladesh, anaemia is estimated to affect 52% of children 6–59 months, with the youngest children (6–23 months) experiencing the highest levels of anaemia (71%). Micronutrient powders (MNPs) are designed to increase micronutrient intake in young children; however, in some settings, the prevalence of anaemia may remain elevated despite the high coverage of MNPs. In a secondary analysis of the Zinc in Powders trial (ZiPT), we identified risk factors that were associated with anaemia among Bangladeshi children 9–11 months of age who received standard 15‐component MNPs, including 10 mg of iron, daily for 24 weeks. At enrolment, socio‐demographic characteristics were collected. Morbidity symptoms were assessed on a semi‐weekly basis. Haemoglobin (measured via single‐drop capillary blood using Hemocue 301+) and child anthropometry were assessed at enrolment and endline (24 weeks). Risk factors for anaemia at endline (24 weeks) were identified using minimally adjusted (age and sex) logistic regression models. Multivariate models were subsequently constructed, controlling for age, sex and significant risk factors. Of the 481 children randomized to the MNP arm, 442 completed the trial and had haemoglobin data available at endline. Anaemia (haemoglobin < 10.5 g/dL) prevalence declined from 54.1% at baseline to 32.6% at endline. In minimally adjusted models, season of enrolment, underweight at enrolment, asset score, hygiene score and frequent morbidity symptoms were associated with the odds of anaemia at endline. However, some factors lost statistical significance in multivariate models. MNPs are an important tool for anaemia prevention; however, they should be part of an integrated approach for anaemia control.

## Summary

- Among young Bangladeshi children who received standard MNPs for 24 weeks, anaemia (haemoglobin < 10.5 mg/dL) prevalence declined from 54% to 33%, still above the World Health Organization's threshold for a public health problem.
- Anaemia at endline (24 weeks) was significantly associated with factors related to poverty, suboptimal hygiene practices, poor growth, season and frequent morbidity in bivariate models, yet some factors lost statistical significance in multivariate models.
- In nutritionally vulnerable settings with a high prevalence of anaemia, MNPs may be insufficient to adequately control anaemia. Multi‐pronged interventions that address the underlying, multifactorial causes of anaemia and integrate prevention and treatment are necessary.

## Graphical abstract

Micronutrient powders (MNPs) are recommended by the World Health Organization to address and prevent anaemia among children in nutritionally vulnerable settings with a high prevalence of anaemia (> 40%); however, following a 24‐week intervention of daily MNP provision among young Bangladeshi children, anaemia was associated with factors related to poverty, suboptimal hygiene practices, poor growth, season and frequent morbidity. This suggests that MNPs may be insufficient to adequately control anaemia, and multi‐pronged interventions that address the underlying, multifactorial causes of anaemia and integrate prevention and treatment are necessary.

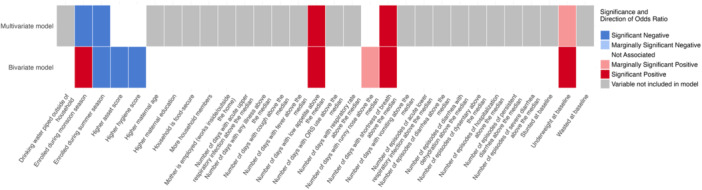

## 1 Introduction

Anaemia affected 40%, or 372 million children under 5 years of age worldwide in 2019 (Stevens et al. 2022), and minimal progress has been made to alleviate the burden of anaemia among young children in recent years (Stevens et al. 2022; World Health Organization 2024b). Anaemia in early childhood can have severe health consequences, including poor immune function, impaired cognitive development and suboptimal physical growth, which may be irreversible and have lasting impacts into adulthood (Hess et al. 2023; Larson et al. 2019). Iron deficiency is considered a major cause of anaemia; however, other micronutrient deficiencies, as well as various environmental, socioeconomic, maternal and genetic risk factors may contribute to the development of anaemia in childhood (Hess et al. 2023).

Meeting the nutritional needs of young children in low‐income settings is challenging due to the limited availability and access to nutrient‐rich foods (Dewey 2013). Since 2011, the World Health Organization (WHO) has recommended the provision of micronutrient powders (MNPs) as an approach to prevent anaemia and iron deficiency among young children 6–23 months by enhancing the micronutrient composition of complementary foods. It is recommended that MNPs be distributed in areas where anaemia prevalence among children under 2 years of age is > 20% (World Health Organization 2016). Standard MNP sachets contain 15 vitamins and minerals, including 10–12.5 mg of iron (World Health Organization 2016). Currently, over 65 countries are implementing MNP programmes as a part of national policies for reducing anaemia and micronutrient deficiencies among young children (Pelletier and DePee 2019).

Although MNPs have successfully decreased the prevalence of anaemia in certain settings (De‐Regil, Jefferds, and Peña‐Rosas 2017; Suchdev et al. 2020), some randomized controlled trials (RCTs) have demonstrated that anaemia prevalence can remain elevated despite MNP provision. In a cluster RCT of daily MNP provision for 6 months among children 6 months of age in Cambodia (N = 925), 77% remained anaemic at endline (Jack et al. 2012). Likewise, in a large RCT in Pakistan, 72% of children 6–23 months (N = 638) who received a daily MNP for 12 months remained anaemic at endline (Soofi et al. 2013). In a large, community‐based cluster RCT of MNP provision on alternating days for 12 months in Burkina Faso, the prevalence of anaemia among children 6–59 months (N = 1318) increased from 75% at baseline to 88% at endline (Lanou et al. 2019).

This suggests that MNPs alone may be inadequate to address anaemia to the greatest extent possible in certain settings. Further evidence is needed to understand key risk factors for anaemia among children who have received MNPs to improve the design and context‐specific targeting of MNP interventions for effective anaemia control.

The recently completed Zinc in Powders trial (ZiPT) evaluated the effects of various doses, forms and frequencies of zinc supplementation, including MNPs, on diarrhoea incidence and linear growth among young children in Dhaka, Bangladesh (Islam et al. 2022a; Islam et al. 2018). Despite high (> 80%) levels of adherence, at the end of the 24‐week intervention period, the prevalence of anaemia among children 9–11 months of age at enrolment who had received standard, 15‐component MNPs containing 10 mg of iron for 24 weeks, was nearly 33% according to the newly revised WHO cutoff (haemoglobin < 10.5 g/dL) and 46% using the previous cutoffs (haemoglobin < 11 g/dL) (Islam et al. 2022a). The objective of this secondary analysis was to identify risk factors for anaemia at endline (24 weeks) among children enroled in the ZiPT trial who had received daily MNPs.

## 2 Methods

### 2.1 Study Design, Setting and Population

The ZiPT trial was a randomized, partially double‐blind, controlled, community‐based efficacy trial conducted in the peri‐urban, low‐income area of Mirpur in Dhaka, Bangladesh. The study protocol has been published previously (Islam et al. 2018). This area was chosen due to its approximation to the International Centre for Diarrheal Disease Research, Bangladesh (icddr,b), large population size and high prevalences of zinc deficiency, diarrhoea and stunting among preschool‐aged children. The trial had six intervention arms with varying doses, forms and/or frequencies of zinc supplementation. A summary of the intervention groups is provided in Supporting Information S1: Table 1. Between October 2017 and July 29, 2019, a total of 5567 children 9–11 months of age were screened for eligibility and 2886 children were enroled in the trial and randomized to one of the six study groups. The analyses presented here are restricted to children who received a standard 15‐component MNP containing 4.1 mg zinc and 10 mg iron (World Health Organization 2016) (manufactured by DSM India Private Limited) that was intended to be consumed daily. The complete composition of the MNP can be found in Supporting Information S1: Table 2.

A detailed description of the study participants has been published previously (Islam et al. 2018). Briefly, children were eligible for enrolment if they had a weight‐for‐length z‐score (WLZ) ≥ −3 according to the 2006 WHO Growth Standards. Children were excluded from the trial if they had any of the following conditions: (1) Severe Acute Malnutrition, defined as WLZ < −3 and/or the presence of bipedal oedema and/or mid‐upper arm circumference (MUAC) < 115 mm; (2) Severe anaemia (haemoglobin concentration < 8 g/dL, as measured by single‐drop capillary blood collected via fingerprick using Hemocue 301+); (3) Congenital anomalies (e.g., cardiac defects, cleft lip or palate) or any other conditions that interfere with feeding and (4) Chromosomal anomalies and other organic problems (e.g., jaundice, tuberculosis, etc.). Children who were identified as severely anaemic (haemoglobin < 8 g/dL) according to Hemocue 301+ measurement of a fingerprick blood sample after 12 weeks of follow‐up were dropped from the study and referred for treatment.

### 2.2 Data Collection

At enrolment, self‐reported data on background, socioeconomic status, education level, household characteristics, sanitation and handwashing practices, source of drinking water, ownership of assets and food security were collected using pretested questionnaires. Certain variables were used to estimate composite household asset scores and hygiene scores. Asset score was estimated using the sum of two subscores: asset score 1, which was the sum of the following possessions: iron, chair/bench, sofa, table, computer, fridge, motorcycle and bank account; asset score 2, which was the sum of the following possessions: electric fan, television, mattress, mobile phone. Asset score could range from 0 to 12. Hygiene score was calculated as the sum of the scores following variables: ‘Wash hands after helping child defecate’; ‘Wash hands before preparing food’, ‘Wash hands after using toilet’ and ‘Uses toilet paper’. Each variable was scored as follows: 1 = never, 2 = rarely, 3 = sometimes, 4 = always. Asset scores could range from 4 to 16. Household food security was assessed using the Household Food Insecurity Access Scale (Coates, Swindale, and Bilinsky 2007).

Trained Field Research Assistants (FRAs) visited the child participant and his/her caregiver at their home twice per week throughout the 24‐week study period. At one of these twice‐weekly visits, the FRA provided the child's caregiver with the MNP for 7 days and instructed the caregiver on proper use. During these home visits, FRAs inquired about MNP consumption over the past 3–4 days, used MNP sachets were collected, and the number consumed was recorded. FRAs also inquired about the presence of diarrhoea, respiratory illness, fever and other morbidity symptoms in the preceding 3–4 days. Definitions of morbidity symptoms can be found in Supporting Information S1: Table 2. If the study child developed diarrhoea, MNPs were temporarily suspended and he/she received standard treatment, including a 20 mg therapeutic zinc supplement in the form of a dispersible tablet for 10 days.

The body weight and length of each child were measured at enrolment, and at 12 and 24 weeks. Study anthropometrists conducted all measurements. Body weight was measured on a balance sensitive to 2 g (SECA, model No. 7281321009, Hamburg, Germany) and length was measured to 0.1 cm using an infantometer (SECA, model No. 4161721009, Hamburg, Germany). The measurements were performed in triplicate following standard procedures (De Onis et al. 2004). Stunting, underweight and wasting were defined as length‐for‐age z‐scores, weight‐for‐age z‐scores and weight‐for‐length z‐scores < −2 SD, respectively (WHO Multicenter Growth Reference Study Group, 2006). Haemoglobin measurements were conducted on all study participants at baseline, 12 weeks, and at the end of the 24‐week intervention period. Haemoglobin concentration was measured from a fingerprick blood sample using a Hemocue, HB 301+ Analyzer (Hemocue AB, Angelholm, Sweden).

### 2.3 Statistical Analysis

The primary outcome variable was anaemia at endline (following the 24‐week intervention period). Recently, the WHO released new guidelines on haemoglobin cutoffs to define anaemia in individuals and populations, which recommend that anaemia be defined as haemoglobin < 10.5 g/dL among children 6–23 months (World Health Organization 2024a). Therefore, anaemia was defined as haemoglobin < 10.5 g/dL for this analysis. However, we also replicated the analyses using the previously recommended cutoff (haemoglobin < 11 g/dL). Analyses were restricted to the children who completed the trial and had haemoglobin concentrations available at endline (24 weeks).

Logistic regression models were used to identify socioeconomic, maternal, environmental and morbidity risk factors that were significantly associated with anaemia at endline in minimally adjusted bivariate models, controlling for child sex and age at enrolment. Multivariable models were subsequently fit including all predictors that were associated with anaemia at endline in bivariate models at a significance level of *p‐*value < 0.1. Because we defined our outcome as anaemia at endline and not change in anaemia status from baseline to endline, we did not control for baseline anaemia status in regression models. However, regression analyses were replicated to include baseline anaemia status and results were reported separately. Collinearity was assessed using variance inflation factors, and if risk factors were significantly correlated, one risk factor was selected for inclusion in the multivariable model to represent the relevant information. If continuous risk factor variables were significantly associated with the outcome, analyses were repeated with categorical variables for ease of interpretation. Results are presented as odds ratios (OR) and 95% confidence intervals (CI). Statistical analyses were performed using R (Version 2023.12.1+402).

## 3 Results

### 3.1 Characteristics of Participant Children and Their Families

Of the 2886 children who were enroled in the ZiPT trial, 481 children were randomized into the standard MNP daily group (Islam et al. 2018). Of these, 442 children completed the trial and had haemoglobin concentration data available at endline (24 weeks). At enrolment, mean ± SD child age was 9.7 ± 0.8 months, and 91% were breastfeeding. Only 23.3% of children were considered healthy at enrolment (had no morbidity symptoms in the past 2 weeks as reported by the child's caregiver). Mean ± SD haemoglobin concentration was 10.4 ± 1.2 g/dL. The prevalences of anaemia according to the recently revised (haemoglobin < 10.5 g/dL) and previously recommended WHO cutoffs (haemoglobin < 11 g/dL) were 54.1% and 68.8%, respectively. Mothers of children enroled in the standard MNP daily group were 24.6 ± 5.4 years of age on average at enrolment. Most (93.2%) women did not work outside the home and completed 6.6 ± 4.1 years of education on average. The mean ± SD household size was 4.5 ± 1.4 people. More than 70% of households had drinking water piped directly into the home, and the mean household hygiene score was considered relatively favourable (12.2 ± 2.4 SD out of 16). Conversely, the mean ± SD asset score was relatively low, 5.8 ± 2.4 out of 12. Nearly 22% of households were classified as food insecure at the time of enrolment (Table 1).

**Table 1:** Enrolment characteristics of children randomized to standard micronutrient powder (MNP) daily group.

	Standard MNP (N = 442)
Child characteristics	
Age (months)	9.74 ± 0.83
Female	51.13% (226)
Breastfeeding	90.95% (402)
Length (cm)	69.30 (2.80)
Weight (kg)	7.93 (1.07)
Length‐for‐age Z‐score	−1.14 ± 1.05
Weight‐for‐age Z‐score	−0.92 ± 1.06
Weight‐for‐length Z‐score	−0.38 ± 1.02
Haemoglobin (g/dL)	10.40 ± 1.16
Anaemic (Hb < 11 g/dL)	68.78% (304)
Anaemic (Hb < 10.5 g/dL^a^)	54.07% (239)
Child is healthy at enrolment	23.30% (103)
Maternal Characteristics	
Age (years)	24.63 ± 5.36
Occupation	
Housewife	93.21% (412)
Work outside/inside the home	6.79% (30)
Years of education completed	6.63 ± 4.14
Socioeconomic Characteristics	
Number of household members	4.50 ± 1.40
Source of drinking water	
Piped into dwelling	72.85% (322)
Piped into yard/plot	22.17% (98)
Other	4.98% (22)
Hygiene score^b^	12.22 ± 2.43
Asset score^c^	5.76 ± 2.37
HFIAS classification	
Food‐secure	78.28% (346)
Food‐insecure	21.72% (96)

*Note:* Values are means ± SD or % (n).

Abbreviations: Hb, haemoglobin; HFIAS, Household Food Insecurity Access Scale.

^a^ Anaemia was estimated using revised WHO cutoffs for children 6–23 months.

^b^ Hygiene score was calculated as the sum of the scores for the following variables: ‘Wash hands after helping child defecate’; ‘Wash hands before preparing food’; ‘Wash hands after using toilet’ and ‘Uses toilet paper’. Each variable was scored as follows: 1 = never, 2 = rarely, 3 = sometimes, 4 = always.

^c^ Asset scores were the sum of two sub‐scores: asset score 1, which was the sum of the following possessions: iron, chair/bench, sofa, table, computer, fridge, motorcycle and bank account; asset score 2, which was the sum of the following possessions: electric fan, television, mattress and mobile phone. Asset score could range from 4 to 16.

### 3.2 Risk Factors for Anaemia Among Children Enroled in the Standard MNP Daily Group at Endline (24 Weeks)

**Figure 1:**
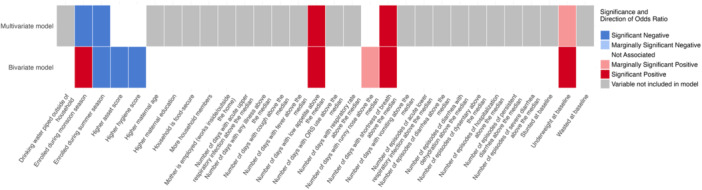
Heatmap of predictors of anaemia (haemoglobin < 10.5 g/dL) among children enroled in the standard MNP daily group^1,2^. ^1^Variables are only shown if they are associated (*p‐*value < 0.1) with anaemia at endline (24 weeks) in either minimally adjusted bivariate or multivariate logistic regression models. ^2^Variables described as lower assume the value of the lower (binary) or lowest variable category. Variables described as higher assume the value of the higher (binary) or highest variable category.

At the end of the 24‐week intervention period, the prevalence estimates of anaemia according to WHO's newly recommended (haemoglobin < 10.5 g/dL) and previously recommended (< 11.0 g/dL) cutoffs were 32.6% and 45.9%, respectively. In bivariate logistic regression models, children living in households with a higher asset score were less likely to be anaemic (*p‐*value for trend = 0.04) at endline following the receipt of standard MNPs daily for 24 weeks (Table 2; Figure 1). Children whose households had a hygiene score above the median had a lower risk of anaemia at endline (OR 0.63, 95% CI 0.42–0.94).

**Table 2:** Predictors of anaemia (haemoglobin < 10.5 g/dL) among children enroled in the standard micronutrient powder (MNP) daily group.

			Minimally adjusted^a^ bivariate model		Multivariate model^b^	
Predictor variable	N	Anaemia prevalence at endline (24 weeks) (%)	OR (95% CI)	*P*‐value	OR (95% CI)	*P‐*value
Maternal age, years						
≤ 20	123	38.21	1	0.22		
21–24	114	32.46	0.76 (0.44–1.30)			
≥ 25	205	29.27	0.66 (0.41–1.06)			
Maternal education						
< 1 year	60	40.00	1	0.13		
2–7 years	197	35.03	0.80 (0.44–1.47)			
≥ 8 years	185	27.57	0.57 (0.31–1.05)			
Maternal occupation						
Housewife	412	32.52	1	0.88		
Employed (Works inside or outside of home)	30	33.33	1.06 (0.46–2.29)			
Household size						
4–5	329	31.91	1	0.63		
6–7	113	34.51	1.12 (0.71–1.75)			
Asset score^c^						
1–3	86	37.21	1	0.04	1	0.15
4–7	243	35.39	0.92 (0.56–1.55)		0.99 (0.58–1.69)	
8–12	113	23.01	0.50 (0.27–0.93)		0.59 (0.30–1.13)	
Hygiene score^d^						
Below median	223	37.34	1	0.02	1	0.28
Above median	209	26.27	0.63 (0.42–0.94)		0.79 (0.51–1.22)	
Drinking water source						
Piped into dwelling	322	30.43	1	0.11		
Piped into yard/plot	98	35.71	1.27 (0.78–2.03)			
Other	22	50.00	2.41 (0.99–5.89)			
Food secure^e^						
No	96	38.54	1	0.17		
Yes	346	30.92	0.72 (0.45–1.16)			
Child age at baseline	442		0.99 (0.78–1.26)	0.92	1.03 (0.79–1.32)	0.84
Child sex						
Male	216	35.19	1	0.25	1	0.49
Female	226	30.09	0.79 (0.53–1.18)		0.86 (0.57–1.31)	
Wasted at baseline^f^						
No	421	32.78	1	0.64		
Yes	21	28.57	0.79 (0.28–2.00)			
Stunted at baseline^g^						
No	352	31.25	1	0.26		
Yes	90	37.78	1.80 (1.06–3.02)			
Underweight at baseline^h^						
No	372	30.38	1	0.03	1	0.10
Yes	70	44.29	1.80 (1.06–3.02)		1.59 (0.91–2.76)	
Season of enrolment						
Winter (November–February)	82	36.79	1	0.03	1	0.03
Summer (February–June)	107	26.24	0.61 (0.37–1.01)		0.55 (0.32–0.95)	
Monsoon (July–October)	106	38.81	1.09 (0.64–1.85)		0.99 (0.57–1.72)	
Number of days with low appetite^i^						
Below the median	329	29.48	1	0.02	1	0.01
Above the median	113	41.49	1.68 (1.08–2.62)		1.94 (1.21–3.13)	
Number of days with acute upper respiratory infection^i^						
Below the median	220	30.00	1	0.29		
Above the median	222	35.14	1.24 (0.83–1.86)			
Number of days with cough^i^						
Below the median	218	35.27	1	0.26		
Above the median	224	29.82	1.26 (0.84–1.89)			
Number of days with any illness^i^						
Below the median	224	31.25	1	0.62		
Above the median	218	33.94	1.11 (0.74–1.66)			
Number of days with runny nose^i^						
Below the median	229	28.38	1	0.05	1	0.48
Above the median	213	37.09	1.48 (0.99–2.22)		1.17 (0.76–1.81)	
Number of days with shortness of breath^i^						
Below the median	424	31.37	1	0.02	1	0.02
Above the median	18	61.11	3.32 (1.28–9.23)		3.36 (1.22–9.79)	
Number of days with elevated respiratory rate^i^						
Below the median	405	32.10	1	0.50		
Above the median	37	37.84	1.27 (0.62–2.55)			
Number of days with vomiting^i^						
Below the median	243	33.74	1	0.52		
Above the median	199	31.16	0.88 (0.58–1.31)			
Number of days with fever^i^						
Below the median	354	31.64	1	0.41		
Above the median	88	36.36	1.23 (0.75–2.00)			
Number of days with ORS use^i^						
Below the median	237	32.07	1	0.83		
Above the median	205	33.17	1.04 (0.70–1.56)			
Number of episodes of acute lower respiratory infection^i^						
Below the median	405	32.10	1	0.50		
Above the median	37	37.84	1.27 (0.62–2.55)			
Number of episodes of diarrhoea^i^						
Below the median	307	30.48	1	0.41		
Above the median	135	34.48	1.18 (0.79–1.77)			
Number of episodes of diarrhoea with dehydration^i^						
Below the median	422	32.46	1	0.81		
Above the median	20	35.00	1.13 (0.41–2.84)			
Number of episodes of diarrhoea with dysentery^i^						
Below the median	420	32.62	1	0.89		
Above the median	22	31.82	0.94 (0.35–2.29)			
Number of episodes of severe diarrhoea^i^						
Below the median	329	31.00	1	0.26		
Above the median	113	37.17	1.29 (0.82–2.02)			
Number of hospital episodes^i^						
Below the median	427	32.32	1	0.59		
Above the median	15	40.00	1.34 (0.44–3.80)			

Abbreviations: CI, Confidence Interval; OR, Odds Ratio; ORS, Oral rehydration salts.

^a^ Minimally adjusted models are adjusted for age at baseline and child sex.

^b^ Multivariate models are adjusted for child age at enrolment, child sex, and all predictor variables that were significantly (*p‐value* < 0.05) associated with anaemia at endline in minimally adjusted models (asset score, hygiene score, underweight at baseline, season of enrolment, number of days with low appetite, runny nose and shortness of breath).

^c^ Hygiene score was calculated as the sum of the scores for the following variables: ‘Wash hands after helping child defecate’; ‘Wash hands before preparing food’; ‘Wash hands after using toilet’ and ‘Uses toilet paper’. Each variable was scored as follows: 1 = never, 2 = rarely, 3 = sometimes, 4 = always.

^d^ Asset scores were the sum of two sub‐scores: asset score 1, which was the sum of the following possessions: iron, chair/bench, sofa, table, computer, fridge, motorcycle and bank account; asset score 2, which was the sum of the following possessions: electric fan, television, mattress and mobile phone. Asset score could range from 4 to 16.

^e^ As defined by the Household Food Insecurity Asset Scale (HFIAS).

^f^ Wasted defined as weight‐for‐length z‐score < −2 standard deviations.

^g^ Stunted defined as length‐for‐age z‐score < −2 standard deviations.

^h^ Underweight defined as weight‐for‐age z‐score < −2.

^i^ Morbidity variable definitions can be found in Supporting Information S1: Table 2.

Children who were underweight at enrolment had a significantly increased risk of anaemia at endline (OR 1.80, CI 1.06–3.02) in bivariate models. Children who were enroled in the summer season were significantly less likely to be anaemic at endline as compared to children enroled in the winter season (OR 0.61, CI 0.37–1.01). Several morbidity symptoms were associated with an increased risk of anaemia at endline, including frequent low appetite (number of days above the median) (OR 1.68, 95% CI 1.08–2.62), shortness of breath (OR 3.32, 95% CI 1.28–9.23) and runny nose (OR 1.48, CI 0.99–2.22) across the 24‐week intervention period. Among the risk factors that were associated with anaemia at endline in bivariate logistic regression models, only season of enrolment, number of days with low appetite and number of days with shortness of breath above the median remained significant in multivariable models (Table 2; Figure 1).

When anaemia was defined by the previously WHO‐recommended cutoffs for children 6–23 months (haemoglobin < 11 g/dL), maternal education (*p‐*value for trend = 0.01), hygiene score (*p‐*value for trend = 0.01) and household food security (OR 0.47, 95% CI 0.30–0.75) were significantly associated with anaemia at endline in bivariate logistic regression models. Household drinking water source (*P‐*value for trend = 0.06) and asset score (*P‐*value for trend = 0.07) were marginally significant in bivariate models. In addition, frequent low appetite (OR 1.60, 95% CI 1.04–2.47), cough (OR 1.46, 95% CI 1.00–2.14) and shortness of breath (OR 3.08, 1.14–9.77) across the 24‐week intervention period were significantly associated with anaemia at endline in bivariate models. However, in multivariate models, only household food security and number of days with shortness of breath remained marginally significant (*p‐*value < 0.1) (Supporting Information: Table 3 and Figure 1). When baseline anaemia status was included in regression models (Supporting Information S1: Table 4 and Figure 3), it was found to be a significant predictor of anaemia at endline in bivariate models (OR 2.90, 1.80–4.83) and multivariate models (OR 2.91, (1.91–4.59). Season of enrolment (*P‐*value for trend = 0.04), frequency with low appetite (OR 2.00, 1.23–3.26) and shortness of breath (OR 2.91, 1.06–8.43) were also significant predictors of anaemia at endline in multivariate models that included baseline anaemia status (Supporting Information S1: Table 4 and Figure 3).

## 4 Discussion

In this secondary analysis of a randomized, partially double‐blind, controlled, community‐based efficacy trial of various forms and frequencies of zinc supplementation, we found many young children in Dhaka, Bangladesh, were anaemic despite receiving standard, 15‐component MNPs containing 10 mg of iron daily for 24 weeks. In bivariate analyses, several socioeconomic, environmental, anthropometric and morbidity risk factors were associated with increased odds of anaemia. However, some risk factor variables lost their statistical significance in multivariate analyses. This is one of the few studies to evaluate risk factors for anaemia among children receiving MNPs.

In the most recent DHS survey in Bangladesh (2011), the national prevalence of anaemia (haemoglobin < 11 g/dL) among children 6–59 months was estimated to be 52%, with the highest prevalence observed among children 6–23 months (71%) (Yusuf et al. 2019). This is comparable to what we observed in our study, suggesting limited progress towards reducing anaemia among young children in recent years in Bangladesh. In the ZiPT trial, we observed a reduction in anaemia prevalence of about 18 percentage points from baseline in the standard MNP group (Islam et al. 2022b). However, we were surprised that nearly 33% (haemoglobin < 10.5 g/dL) [46% (haemoglobin < 11 g/dL)] of children were anaemic at endline despite the provision of MNPs daily for nearly 6 months and high adherence (81.7%). This is twice the WHO‐recommended frequency of MNP provision (90 sachets delivered over a 6‐month period, or one sachet every other day). This suggests that anaemia remained a significant public health problem, as defined by the WHO as an anaemia prevalence of > 20% among children 6–23 months.

It is not completely unexpected that anaemia rates may remain high; anaemia is a multifactorial issue, and the causes of anaemia have been described as a complex ecology of internal and external factors (Raiten et al. 2023). MNPs were designed to prevent the nutritional causes of anaemia by meeting children's daily micronutrient needs; however, MNPs do not address non‐nutritional causes (Chaparro and Suchdev 2019; Raiten et al. 2023). Consistent with conceptual models of anaemia and previous studies in South Asia (Chaparro and Suchdev 2019; Engle‐Stone et al. 2017; Kundu et al. 2023), in bivariate models we observed that children who had an increased risk of anaemia resided in households with lower socioeconomic status. Poor socioeconomic status is associated with an increased risk of anaemia through multiple pathways, such as poor water, sanitation and hygiene practices and more limited intake of nutrient‐dense foods (Hess et al. 2023). In bivariate models, poor household hygiene practices were associated with anaemia. Furthermore, children living in poor sanitary conditions are more likely to experience more morbidity and mortality (Victora et al. 2022), and we observed that frequent morbidity symptoms were associated with increased odds of anaemia in this study. Infection increases inflammation, which triggers the release of hepcidin into circulation, which can reduce the absorption of iron and limit the mobilization of iron from body tissues (Brittenham et al. 2023; Ganz 2018). Children who have frequent illnesses are also likely to experience undernutrition and poor growth (Dewey and Mayers 2011), and we observed that children who were underweight at enrolment were more likely to be anaemic at endline.

In multivariate models, many correlates lost their statistically significant association with anaemia. This may be explained by the complex interactions between the various underlying risk factors of anaemia, as the most vulnerable children are likely to experience several risk factors simultaneously (Balarajan et al. 2011; Hess et al. 2023). Therefore, although we assessed collinearity, associations with anaemia were likely influenced by other underlying and unmeasured risk factor variables in the multivariate models.

It is important to note that baseline anaemia status was also a significant risk factor for anaemia at endline (Supporting Information S1: Table 4 and Figure 3). We did not include baseline anaemia as a predictor in our primary regression analyses. Because MNP programmes are intended to be initiated without assessment of anaemia status at the individual level before the intervention is initiated, our goal was to identify the predictors of anaemia among children who had received the MNP intervention irrespective of their baseline anaemia status. However, we note that when baseline anaemia was included as a predictor in multivariate models, the strength and direction of associations of other predictors with anaemia at endline did not change appreciably (Supporting Information S1: Table 4).

Although micronutrient biomarkers were unavailable for all children in the ZiPT study, micronutrient status was measured in a small subset of 58 children enroled in the standard MNP group (Islam et al. 2022b). At baseline, the prevalence of IDA (anaemia defined as haemoglobin < 10.5 g/dL) and inflammation‐adjusted low serum ferritin (ferritin < 12/ugL) among these children was 47%, and at endline, the prevalence of IDA was 16% (Supporting Information S1: Table 5). This suggests that that MNPs were important for reducing the prevalence of IDA from baseline to endline; however, a considerable proportion of anaemia at endline may have been due to remaining iron deficiency. IDA is especially common among children 6–23 months, whose iron stores deplete rapidly after birth and iron needs increase substantially during the first 2 years of life (Lönnerdal, Georgieff, and Hernell 2015).

Our findings highlight that children who experience multiple risk factors for anaemia likely need more than MNPs alone. We note that in the control group in the main trial, where children received a placebo powder for 24 weeks, anaemia (haemoglobin < 11 g/dL) prevalence was 75% (haemoglobin < 10.5 g/dL; 62%) at endline (24 weeks) compared to a baseline prevalence of 72% (haemoglobin < 10.5 g/dL; 56%) (Islam et al. 2022b). Thus, anaemia prevalence persisted over time in the absence of any intervention. This suggests that MNPs were a critical tool for anaemia reduction and/or prevention in this population. This also demonstrates the importance of targeting MNP programmes to the children at greatest risk and considering multi‐sectoral interventions to address underlying causes of anaemia to further reduce anaemia in this context. For example, in this study, vulnerable children may have benefited from additional interventions to improve hygiene practices and infectious disease control (World Health Organization 2017) alongside the provision of MNPs. Furthermore, targeting these interventions among children who were underweight prior to the intervention may have been important for improving anaemia outcomes. In addition, prioritizing initiation of MNP provision in the summer months (February to June) season as opposed to the winter (November to February) or Monsoon season (July to October) may have improved anaemia outcomes in this setting. This is consistent with evidence suggesting there is a higher prevalence of household food insecurity in the *boro* harvest season (April‐June) in Bangladesh (Raihan et al. 2018).

In addition, although children who experienced severe anaemia (haemoglobin < 8 g/dL) were referred for further diagnosis and treatment in this study, children who experienced any anaemia may have benefitted from additional screening for iron deficiency and potential treatment for IDA (Baker and Greer 2010; Mattiello et al. 2020). The WHO indicates that the use of MNPs is intended to be a preventative strategy that is implemented at the population level without screening for any condition or disease (World Health Organization 2016), as laboratory testing used for diagnostic screening is often impractical in programmatic or field settings. However, MNPs provided only 10 mg of iron, and this dose is insufficient to treat IDA (World Health Organization 2001). Identifying children with IDA may be critical to successfully and sustainably reducing rates of anaemia by repleting iron stores via treatment with therapeutic iron supplementation. In addition, interventions aimed towards dietary modification to improve iron intake should be considered, alongside MNP provision, to promote improvement in iron status. Furthermore, as noted above, these approaches should ideally be combined with infectious disease control so that iron may be absorbed and mobilized from tissues (Brittenham et al. 2023).

In this study, we found that a large proportion (13%) of children were no longer considered anaemic when the cutoff shifted from 11 to 10.5 g/dL (Supporting Information S1: Figure 1). The WHO recently updated its guidelines for anaemia cutoffs to suggest separate cutoffs for the 6–23 months age group based on consistently reported differences in haemoglobin concentrations among children 6–23 months versus children 24–59 months, as well as differences in nutrient requirements between these two age strata, along with other rationales (World Health Organization 2024a). The updated cutoffs were derived from the fifth percentiles of haemoglobin distributions derived from apparently healthy populations in the USA, Canada, Ecuador and Bangladesh (Braat et al. 2024). Among the studies used to inform the guidelines, haemoglobin was measured via venous blood and using a haematology analyser, and we note that the guidelines do not provide adjustments for haemoglobin using single‐drop capillary blood using a Hemocue instrument. Very few studies to date have used these new haemoglobin cutoffs for this age group, and we consider this a strength of our analysis. We note that household food security and maternal education were significant predictors of anaemia when classified as haemoglobin < 11 g/dL (Supporting Information S1: Table 3 and Figure 2); however, these variables were non‐significantly associated with anaemia when classified as haemoglobin < 10.5 g/dL in bivariate models.

Additional strengths of this study include the large sample size, the randomized, controlled design, and rigorous data on adherence. In addition, we collected frequent, continuous data on morbidity symptoms throughout the intervention period. However, we acknowledge that this secondary analysis had several limitations. While we included a wide range of risk factors in our analysis, we did not assess the primary causes of anaemia, including micronutrient and inflammatory biomarkers, data on genetic hemoglobinopathies and other red blood cell disorders, and infections such as soil‐transmitted helminthiasis. We also did not capture data on dietary intake or complementary feeding practices. Finally, we were limited to haemoglobin measurements using single‐drop capillary blood collected via fingerprick and measured using a HemoCue 301+ device. Although this method of haemoglobin measurement is frequently used in field settings, our estimates of anaemia may have been subject to measurement error, resulting in inaccurate estimates of anaemia (Hackl et al. 2024). The gold‐standard method of haemoglobin measurement is the assessment of venous blood using an automated haematology analyser. This method is challenging in field contexts, as well as costly, and there is no consensus regarding the acceptable level of error for population‐based assessments performed using single‐drop capillary blood and a point‐of‐care hemoglobinometer. However, using venous blood in a point‐of‐care hemoglobinometer may introduce less measurement error. We acknowledge this as a limitation of our analysis, and future studies should consider the use of venous blood instead of single‐drop capillary blood where possible (Karakochuk et al. 2024).

In conclusion, this study allowed us to evaluate modifiable risk factors among children who were vulnerable to anaemia regardless of MNP provision. Anaemia is a complex condition that requires coordinated efforts to effectively and sustainably reduce it (Loechl et al. 2023). Although MNPs are an important tool for anaemia prevention, in isolation, they are insufficient to resolve anaemia completely and should be part of an integrated and comprehensive approach for anaemia control. This study highlighted areas for implementation of co‐interventions that, together with MNPs, may more effectively manage anaemia among vulnerable children in this region in Bangladesh. Future studies that leverage multiple data sources, including biomarker data, to elucidate context‐specific causes and underlying determinants anaemia will be important for MNP programme planning.

## Author Contributions

Lauren Thompson, Julie M. Long, Jamie L. E. Westcott, M. Munirul Islam, Robert E. Black, Nancy F. Krebs and Christine M. McDonald designed and conducted the study. Lauren Thompson, Charles Arnold and Janet Peerson performed the data analysis. Lauren Thompson wrote the initial draft of the manuscript. All authors contributed to manuscript revisions. All authors read and approved the final manuscript.

## Ethics Statement

Ethical approval for ZiPT was provided by the Institutional Review Boards of icddr,b, Children's Hospital Oakland Research Institute, and the University of Colorado, Denver. The research protocol was registered at clinicaltrials.gov as NCT03406793 and previously published (Islam et al. 2018). Informed consent was provided by the child's mother or primary caregiver in Bangla, the national language of Bangladesh.

## Conflicts of Interest

The authors declare no conflicts of interest.

## Supporting information

Supporting information.

## Data Availability

The data that support the findings of this study are available on request from the corresponding author. The data are not publicly available due to privacy or ethical restrictions.
