# Supplementary material for: Predictors of Anaemia Among Young Children Receiving Daily Micronutrient Powders (MNPs) for 24 Weeks in Bangladesh: A Secondary Analysis of the Zinc in Powders Trial
Source: Matern Child Nutr. 2025 Feb 10;21(3):e13806. doi: 10.1111/mcn.13806 (PMC12150132; doi:10.1111/mcn.13806)
Supplement: Supplementary file 1 — Supporting information. [file MCN-21-e13806-s001.docx]

| Study Group | Description | Form | Micronutrient Content | Frequency of Supplementation |
| --- | --- | --- | --- | --- |
| 1 | Standard 15-component MNP | Powder | Vitamin A: 400 μg Vitamin D: 5 μg Vitamin E: 5 mg Vitamin C: 30 mg Thiamine: 0.5 mg Riboflavin: 0.5 mg Niacin: 6 mg Pyridoxine: 0.5 mg Vitamin B12: 0.9 mg Folate: 150 μg Iron: 10 mg Zinc: 4.1 mg Copper: 0.56 mg Selenium: 17.0 μg Iodine: 90 μg | Daily for 24 weeks |
| 2 | High zinc, low iron MNP | Powder | Same as study group 1, except with 10 mg zinc and 6 mg iron | Daily for 24 weeks |
| 3 | High zinc, low iron MNP; high-zinc, no-iron MNP on alternating days | Powder | High zinc, low iron MNP; high-zinc, no-iron MNP on alternating days | Daily for 24 weeks |
| 4 | Dispersible zinc supplement | Dispersible tablet | 10 mg zinc | Daily for 24 weeks |
| 5 | Intermittent zinc supplement | Dispersible tablet | 10 mg zinc | Daily for 14 days at enrolment and 3 months, placebo tablet on all other days |
| 6 | Placebo powder | Powder | None | Daily for 24 weeks |

**Supplementary Table 1**: Characteristics of Zinc in Powders Trial (ZiPT) Study Interventions

**Supplementary Table 2:** Definitions of Morbidity Variables

| **Morbidity Variable** | **Definition** |
| --- | --- |
| Diarrhea | Any three or more loose, liquid, or watery stools over a 24-hour period, separated in time from an earlier or subsequent episode for at least 2 consecutive diarrhea-free days since last visit (yes/no) and number of days/episodes. |
| Severe diarrhea | Severe diarrhea was defined as an episode of diarrhea with dehydration, hospitalization (for any reason), or 6 or more non-bloody loose stools in a single day occurring during the episode since last visit (yes/no) and number of days/episodes. |
| Dysentery | Any diarrheal episode in which the loose or watery stools contain visible blood since last visit (yes/no) and number of days/episodes. |
| Diarrhea with dehydration | Diarrhea with dehydration was defined as an episode of diarrhea in which dehydration (reported) occurred at least once since last visit (yes/no) and number of days/episodes. |
| Fever | Axillary temperature above 38.3°C reported either by the caregiver or the study workers, who carried thermometers, since last visit (yes/no) and number of days. |
| Acute upper respiratory infection | Any pharyngitis or rhinitis, both without rapid respiratory rate or chest indrawing since last visit (yes/no) and number of days. |
| Acute lower respiratory infection | Any cough or difficulty breathing, rapid respiratory rate (>50 breaths/minute in infants 9–11 months of age and >40 breaths per minute among infants 12 months of age and older), and either a fever of >38.3°C or chest retractions since last visit (yes/no) and number of days. |
| Hospitalization | Any overnight stay in the hospital due to illness since last visit (yes/no) and number of days. |
| Cough | Any cough since last visit (yes/no) and number of days. |
| Vomiting | Any vomiting since last visit (yes/no) and number of days. |
| Runny nose | Any runny nose since last visit (yes/no) and number of days. |
| Low appetite | Any low appetite since last visit (yes/no) and number of days. |
| Skin rash | Any skin rash since last visit (yes/no) and number of days. |
| Shortness of breath | Any shortness of breath since last visit (yes/no) and number of days. |
| Elevated respiratory rate | Rapid respiratory rate (>50 breaths/minute in infants 9–11 months of age and >40 breaths per minute among infants 12 months of age and older) since last visit (yes/no) and number of days. |
| Any illness | Any morbidity since last visit (yes/no) and number of days (including persistent diarrhea, severe diarrhea, diarrhea with dehydration, dysentery, acute lower respiratory infection, hospitalization, fever, cough, vomiting, runny nose, any illness, low appetite, skin rash, acute upper respiratory infection). |

**Supplementary Table 3:** Predictors of anemia (hemoglobin <11 g/dL) among children enrolled in the standard micronutrient powder (MNP) daily group

|  |  |  | Minimally-Adjusted^1^ Models | | Multivariate Model | |
| --- | --- | --- | --- | --- | --- | --- |
| Predictor Variable | **N** | **Anemia Prevalence at Endline (24 Weeks) (%)** | **OR (95% CI)** | **P-Value** | **OR (95% CI)** | **P-Value** |
| Maternal age (years) |  |  |  |  |  |  |
| ≤20 | 123 | 50.41 | 1 | 0.32 |  |  |
| 21-24 | 114 | 47.37 | 0.88 (0.52-1.47) |  |  |  |
| ≥25 | 205 | 42.44 | 0.71 (0.45-1.12) |  |  |  |
|  |  |  |  |  |  |  |
| Maternal education |  |  |  |  |  |  |
| <1 year | 60 | 58.33 | 1 | **0.01** | 1 | 0.28 |
| 2-7 years | 197 | 49.75 | 0.69 (0.38-1.24) |  | 0.84 (0.55-1.28) |  |
| ≥8 years | 185 | 37.84 | 0.43 (0.23-0.77) |  |  |  |
|  |  |  |  |  |  |  |
| Maternal occupation |  |  |  |  |  |  |
| Housewife | 412 | 46.36 | 1 | 0.56 |  |  |
| Employed (Works inside or outside of home) | 30 | 40.00 | 0.80 (0.36-1.69) |  |  |  |
|  |  |  |  |  |  |  |
| Household size |  |  |  |  |  |  |
| 4-5 | 329 | 45.90 | 1 | 1.00 |  |  |
| 6-7 | 113 | 46.02 | 1.00 (0.65-1.54) |  |  |  |
|  |  |  |  |  |  |  |
| Asset Score^2^ |  |  |  |  |  |  |
| 1-3 | 86 | 50.00 | 1 | **0.06** | 1 | 0.52 |
| 4-7 | 243 | 48.97 | 0.96 (0.59-1.58) |  | 1.14 (0.68-1.93) |  |
| 8-12 | 113 | 36.28 | 0.57 (0.32-1.01) |  | 0.086 (0.45-1.65) |  |
|  |  |  |  |  |  |  |
| Hygiene Score^3^ |  |  |  |  |  |  |
| Below median | 223 | 51.93 | 1 | **0.01** | 1 | 0.42 |
| Above median | 209 | 39.23 | 0.60 (0.41-0.88) |  | 0.84 (0.55-1.28) |  |
|  |  |  |  |  |  |  |
|  |  |  |  |  |  |  |
| Drinking Water Source |  |  |  |  |  |  |
| Piped into dwelling | 322 | 43.17 | 1 | **0.07** | 1 | 0.47 |
| Piped into yard/plot | 98 | 51.02 | 1.36 (0.86-2.14) |  | 1.07 (0.66-1.74) |  |
| Other | 22 | 63.64 | 2.53 (1.04-6.56) |  | 1.80 (0.71-4.85) |  |
|  |  |  |  |  |  |  |
| Food Secure^4^ |  |  |  |  |  |  |
| No | 96 | 60.42 | 1 | **0.00** | 1 | **0.09** |
| Yes | 346 | 41.91 | 0.47 (0.30-0.75) |  | 0.64 (0.38-1.07) |  |
|  |  |  |  |  |  |  |
| Child age at baseline | 442 |  | 0.99 (0.78-1.26) | 0.92 | 0.89 (0.70-1.14) | 0.37 |
|  |  |  |  |  |  |  |
| Child sex |  |  |  |  |  |  |
| Male | 216 | 48.61 | 1 | 0.79 | 1 | 0.47 |
| Female | 226 | 43.36 | 0.79 (0.53-1.18) |  | 0.87 (0.58-1.28) |  |
|  |  |  |  |  |  |  |
| Wasted at baseline^5^ |  |  |  |  |  |  |
| No | 421 | 46.32 | 1 | 0.42 |  |  |
| Yes | 21 | 38.10 | 0.69 (0.27-1.67) |  |  |  |
|  |  |  |  |  |  |  |
| Stunted at baseline^6^ |  |  |  |  |  |  |
| No | 352 | 44.32 | 1 | 0.19 |  |  |
| Yes | 90 | 52.22 | 1.37 (0.86-2.19) |  |  |  |
|  |  |  |  |  |  |  |
| Underweight at baseline^7^ |  |  |  |  |  |  |
| No | 372 | 44.09 | 1 | **0.08** | 1 | 0.30 |
| Yes | 70 | 55.71 | 1.58 (0.95-2.67) |  | 1.33 (0.77-2.33) |  |
|  |  |  |  |  |  |  |
| Season of enrollment |  |  |  |  |  |  |
| Winter | 82 | 47.17 | 1 | 0.21 |  |  |
| Summer | 107 | 41.58 | 0.80 (0.50-1.28) |  |  |  |
| Monsoon | 106 | 51.49 | 1.18 (0.71-1.97) |  |  |  |

|  |  |  | Minimally-Adjusted^1^ Models | | Multivariate Model | |
| --- | --- | --- | --- | --- | --- | --- |
| Predictor Variable | **N** | **Anemia Prevalence at Endline (24 Weeks) (%)** | **OR (95% CI)** | **P-Value** | **OR (95% CI)** | **P-Value** |
| Number of days with low appetite^8^ |  |  |  |  |  |  |
| Below the median | 329 | 42.86 | 1 | **0.03** | 1 | **0.05** |
| Above the median | 113 | 54.87 | 1.60 (1.04-2.47) |  | 1.57 (0.99-2.48) |  |
|  |  |  |  |  |  |  |
| Number of days with acute upper respiratory infection^8^ |  |  |  |  |  |  |
| Below the median | 220 | 41.82 | 1 | 0.51 |  |  |
| Above the median | 222 | 50.00 | 1.35 (0.93-1.98) |  |  |  |
|  |  |  |  |  |  |  |
| Number of days with cough^8^ |  |  |  |  |  |  |
| Below the median | 218 | 40.83 | 1 | **0.05** | 1 | 0.52 |
| Above the median | 224 | 50.89 | 1.46 (1.00-2.14) |  | 1.16 (0.74-1.80) |  |
|  |  |  |  |  |  |  |
| Number of days with any illness^8^ |  |  |  |  |  |  |
| Below the median | 224 | 42.86 | 1 | 0.27 |  |  |
| Above the median | 218 | 49.08 | 1.24 (0.85-1.82) |  |  |  |
|  |  |  |  |  |  |  |
| Number of days with runny nose^8^ |  |  |  |  |  |  |
| Below the median | 229 | 41.48 | 1 | **0.07** | 1 | 0.43 |
| Above the median | 213 | 50.70 | 1.42 (0.98-2.08) |  | 1.19 (0.77-1.84) |  |
|  |  |  |  |  |  |  |
| Number of days with shortness of breath^8^ |  |  |  |  |  |  |
| Below the median | 424 | 44.81 | 1 | **0.04** | 1 | **0.10** |
| Above the median | 18 | 72.22 | 3.08 (1.14-9.77) |  | 2.48 (0.88-8.13) |  |
|  |  |  |  |  |  |  |
| Number of days with elevated respiratory rate^8^ |  |  |  |  |  |  |
| Below the median | 405 | 45.43 | 1 | 0.45 |  |  |
| Above the median | 37 | 51.35 | 1.30 (0.66-2.59) |  |  |  |
|  |  |  |  |  |  |  |
| Number of days with vomiting^8^ |  |  |  |  |  |  |
| Below the median | 243 | 46.91 | 1 | 0.60 |  |  |
| Above the median | 199 | 44.72 | 0.90 (0.62-1.32) |  |  |  |
|  |  |  |  |  |  |  |
| Number of days with fever^8^ |  |  |  |  |  |  |
| Below the median | 354 | 45.76 | 1 | 0.97 |  |  |
| Above the median | 88 | 46.59 | 0.99 (0.66-1.50) |  |  |  |
|  |  |  |  |  |  |  |
| Number of days with ORS use^8^ |  |  |  |  |  |  |
| Below the median | 237 | 43.04 | 1 | 0.21 |  |  |
| Above the median | 205 | 49.27 | 1.27 (0.87-1.85) |  |  |  |
|  |  |  |  |  |  |  |
| Number of episodes of acute lower respiratory infection^8^ |  |  |  |  |  |  |
| Below the median | 405 | 45.43 | 1 | 0.45 |  |  |
| Above the median | 37 | 51.35 | 1.30 (0.66-2.59) |  |  |  |
|  |  |  |  |  |  |  |
| Number of episodes of diarrhea^8^ |  |  |  |  |  |  |
| Below the median | 307 | 42.38 | 1 | 0.19 |  |  |
| Above the median | 135 | 49.14 | 1.29 (0.88-1.88) |  |  |  |
|  |  |  |  |  |  |  |
| Number of episodes of diarrhea with dehydration^8^ |  |  |  |  |  |  |
| Below the median | 422 | 45.26 | 1 | 0.23 |  |  |
| Above the median | 20 | 60.00 | 1.76 (0.71-4.60) |  |  |  |
|  |  |  |  |  |  |  |
| Number of episodes of diarrhea with dysentery^8^ |  |  |  |  |  |  |
| Below the median | 420 | 45.95 | 1 | 0.91 |  |  |
| Above the median | 22 | 45.45 | 0.95 (0.39-2.26) |  |  |  |
|  |  |  |  |  |  |  |
| Number of episodes of severe diarrhea^8^ |  |  |  |  |  |  |
| Below the median | 329 | 44.98 | 1 | 0.57 |  |  |
| Above the median | 113 | 48.67 | 1.13 (0.74-1.74) |  |  |  |
|  |  |  |  |  |  |  |
| Number of hospital episodes^8^ |  |  |  |  |  |  |
| Below the median | 427 | 45.90 | 1 | 0.96 |  |  |
| Above the median | 15 | 46.67 | 0.97 (0.33-2.76) |  |  |  |

Abbreviations: OR; Odds Ratio, CI; Confidence Interval, ORS; Oral rehydration salts.

^1^Minimally-adjusted models are adjusted for age at baseline and child sex. ^2^Hygiene score was calculated as the sum of the scores for the following variables: “Wash hands after helping child defecate”; “Wash hands before preparing food”; “Wash hands after using toilet”; and “Uses toilet paper”. Each variable was scored as follows: 1 = never, 2 = rarely, 3 = sometimes, 4 = always. ^3^Asset scores were the sum of two sub-scores: asset score 1, which was the sum of the following possessions: iron, chair/bench, sofa, table, computer, fridge, motorcycle, and bank account; asset score 2, which was the sum of the following possessions: electric fan, television, mattress, mobile phone. Asset score could range from 4 to 16. ^4^As defined by the Household Food Insecurity Asset Scale (HFIAS). ^5^Wasted defined as weight-for-length z-score <-2 standard deviations. ^6^Stunted defined as length-for-age z-score <-2 standard deviations. ^7^Underweight defined as weight-for-age z-score <-2. ^8^Morbidity variable definitions can be found Supporting Information: Table 2

**Supplementary Table 4:** Predictors of anemia (hemoglobin <10.5 g/dL) among children enrolled in the standard micronutrient powder (MNP) daily group including baseline anemia status as a predictor

|  |  |  | Minimally-adjusted^1^ bivariate model | | Multivariate model | |
| --- | --- | --- | --- | --- | --- | --- |
| Predictor variable | **N** | **Anemia prevalence at endline (24 weeks) (%)** | **OR (95% CI)** | **P-Value** | **OR (95% CI)** | **P-Value** |
| Maternal age, years |  |  |  |  |  |  |
| ≤20 | 123 | 38.21 | 1 | 0.22 |  |  |
| 21-24 | 114 | 32.46 | 0.76 (0.44-1.30) |  |  |  |
| ≥25 | 205 | 29.27 | 0.66 (0.41-1.06) |  |  |  |
|  |  |  |  |  |  |  |
| Maternal education |  |  |  |  |  |  |
| <1 year | 60 | 40.00 | 1 | 0.13 |  |  |
| 2-7 years | 197 | 35.03 | 0.80 (0.44-1.47) |  |  |  |
| ≥8 years | 185 | 27.57 | 0.57 (0.31-1.05) |  |  |  |
|  |  |  |  |  |  |  |
| Maternal occupation |  |  |  |  |  |  |
| Housewife | 412 | 32.52 | 1 | 0.88 |  |  |
| Employed (Works inside or outside of home) | 30 | 33.33 | 1.06 (0.46-2.29) |  |  |  |
|  |  |  |  |  |  |  |
| Household size |  |  |  |  |  |  |
| 4-5 | 329 | 31.91 | 1 | 0.63 |  |  |
| 6-7 | 113 | 34.51 | 1.12 (0.71-1.75) |  |  |  |
|  |  |  |  |  |  |  |
| Asset Score^2^ |  |  |  |  |  |  |
| 1-3 | 86 | 37.21 | 1 | **0.04** | 1 | 0.46 |
| 4-7 | 243 | 35.39 | 0.92 (0.56-1.55) |  | 0.99 (0.58-1.72) |  |
| 8-12 | 113 | 23.01 | 0.50 (0.27-0.93) |  | 0.55 (0.29-1.15) |  |
|  |  |  |  |  |  |  |
| Hygiene Score^3^ |  |  |  |  |  |  |
| Below median | 223 | 37.34 | 1 | **0.02** | 1 | 0.46 |
| Above median | 209 | 26.27 | 0.63 (0.42-0.94) |  | 0.84 (0.51-1.33) |  |
|  |  |  |  |  |  |  |
|  |  |  |  |  |  |  |
| Drinking Water Source |  |  |  |  |  |  |
| Piped into dwelling | 322 | 30.43 | 1 | 0.11 |  |  |
| Piped into yard/plot | 98 | 35.71 | 1.27 (0.78-2.03) |  |  |  |
| Other | 22 | 50.00 | 2.41 (0.99-5.89) |  |  |  |
|  |  |  |  |  |  |  |
| Food Secure^4^ |  |  |  |  |  |  |
| No | 96 | 38.54 | 1 | 0.17 |  |  |
| Yes | 346 | 30.92 | 0.72 (0.45-1.16) |  |  |  |
|  |  |  |  |  |  |  |
| Child age at baseline | 442 |  | 0.99 (0.78-1.26) | 0.92 | 0.97 (0.75-1.26) | 0.83 |
|  |  |  |  |  |  |  |
| Anemic at baseline (Hb < 10.5 g/dL) |  |  |  |  |  |  |
| No | 138 | 62.08 | 1 | **0.00** | 1 | **0.00** |
| Yes | 304 | 82.64 | 2.90 (1.80-4.83) |  | 2.91 (1.91-4.59) |  |
|  |  |  |  |  |  |  |
| Child sex |  |  |  |  |  |  |
| Male | 216 | 35.19 | 1 | 0.25 | 1 | 0.61 |
| Female | 226 | 30.09 | 0.79 (0.53-1.18) |  | 0.89 (0.58-1.37) |  |
|  |  |  |  |  |  |  |
| Wasted at baseline^5^ |  |  |  |  |  |  |
| No | 421 | 32.78 | 1 | 0.64 |  |  |
| Yes | 21 | 28.57 | 0.79 (0.28-2.00) |  |  |  |
|  |  |  |  |  |  |  |
| Stunted at baseline^6^ |  |  |  |  |  |  |
| No | 352 | 31.25 | 1 | 0.26 |  |  |
| Yes | 90 | 37.78 | 1.80 (1.06-3.02) |  |  |  |
|  |  |  |  |  |  |  |
| Underweight at baseline^7^ |  |  |  |  |  |  |
| No | 372 | 30.38 | 1 | **0.03** | 1 | 0.12 |
| Yes | 70 | 44.29 | 1.80 (1.06-3.02) |  | 1.58 (0.89-2.78) |  |
|  |  |  |  |  |  |  |
| Season of enrollment |  |  |  |  |  |  |
| Winter | 82 | 36.79 | 1 | **0.03** | 1 | **0.04** |
| Summer | 107 | 26.24 | 0.61 (0.37-1.01) |  | 0.59 (0.33-1.03) |  |
| Monsoon | 106 | 38.81 | 1.09 (0.64-1.85) |  | 1.10 (0.62-1.94) |  |

|  |  |  | Minimally-adjusted bivariate model | | Multivariate model | |
| --- | --- | --- | --- | --- | --- | --- |
| Predictor Variable | **N** | **Anemia prevalence at endline**  **(24 weeks) (%)** | **OR (95% CI)** | **P-Value** | **OR (95% CI)** | **P-Value** |
| Number of days with low appetite^8^ |  |  |  |  |  |  |
| Below the median | 329 | 29.48 | 1 | **0.02** | 1 | **0.01** |
| Above the median | 113 | 41.49 | 1.68 (1.08-2.62) |  | 2.00 (1.23-3.26) |  |
|  |  |  |  |  |  |  |
| Number of days with acute upper respiratory infection^8^ |  |  |  |  |  |  |
| Below the median | 220 | 30.00 | 1 | 0.29 |  |  |
| Above the median | 222 | 35.14 | 1.24 (0.83-1.86) |  |  |  |
|  |  |  |  |  |  |  |
| Number of days with cough^8^ |  |  |  |  |  |  |
| Below the median | 218 | 35.27 | 1 | 0.26 |  |  |
| Above the median | 224 | 29.82 | 1.26 (0.84-1.89) |  |  |  |
|  |  |  |  |  |  |  |
| Number of days with any illness^8^ |  |  |  |  |  |  |
| Below the median | 224 | 31.25 | 1 | 0.62 |  |  |
| Above the median | 218 | 33.94 | 1.11 (0.74-1.66) |  |  |  |
|  |  |  |  |  |  |  |
| Number of days with runny nose^8^ |  |  |  |  |  |  |
| Below the median | 229 | 28.38 | 1 | **0.05** | 1 | 0.47 |
| Above the median | 213 | 37.09 | 1.48 (0.99-2.22) |  | 1.18 (0.75-1.85) |  |
|  |  |  |  |  |  |  |
| Number of days with shortness of breath^8^ |  |  |  |  |  |  |
| Below the median | 424 | 31.37 | 1 | **0.02** | 1 | **0.04** |
| Above the median | 18 | 61.11 | 3.32 (1.28-9.23) |  | 2.91 (1.06-8.43) |  |
|  |  |  |  |  |  |  |
| Number of days with elevated respiratory rate^8^ |  |  |  |  |  |  |
| Below the median | 405 | 32.10 | 1 | 0.50 |  |  |
| Above the median | 37 | 37.84 | 1.27 (0.62-2.55) |  |  |  |
|  |  |  |  |  |  |  |
| Number of days with vomiting^8^ |  |  |  |  |  |  |
| Below the median | 243 | 33.74 | 1 | 0.52 |  |  |
| Above the median | 199 | 31.16 | 0.88 (0.58-1.31) |  |  |  |
|  |  |  |  |  |  |  |
| Number of days with fever^8^ |  |  |  |  |  |  |
| Below the median | 354 | 31.64 | 1 | 0.41 |  |  |
| Above the median | 88 | 36.36 | 1.23 (0.75-2.00) |  |  |  |
|  |  |  |  |  |  |  |
| Number of days with ORS use^8^ |  |  |  |  |  |  |
| Below the median | 237 | 32.07 | 1 | 0.83 |  |  |
| Above the median | 205 | 33.17 | 1.04 (0.70-1.56) |  |  |  |
|  |  |  |  |  |  |  |
| Number of episodes of acute lower respiratory infection^8^ |  |  |  |  |  |  |
| Below the median | 405 | 32.10 | 1 | 0.50 |  |  |
| Above the median | 37 | 37.84 | 1.27 (0.62-2.55) |  |  |  |
|  |  |  |  |  |  |  |
| Number of episodes of diarrhea^8^ |  |  |  |  |  |  |
| Below the median | 307 | 30.48 | 1 | 0.41 |  |  |
| Above the median | 135 | 34.48 | 1.18 (0.79-1.77) |  |  |  |
|  |  |  |  |  |  |  |
| Number of episodes of diarrhea with dehydration^8^ |  |  |  |  |  |  |
| Below the median | 422 | 32.46 | 1 | 0.81 |  |  |
| Above the median | 20 | 35.00 | 1.13 (0.41-2.84) |  |  |  |
|  |  |  |  |  |  |  |
| Number of episodes of diarrhea with dysentery^8^ |  |  |  |  |  |  |
| Below the median | 420 | 32.62 | 1 | 0.89 |  |  |
| Above the median | 22 | 31.82 | 0.94 (0.35-2.29) |  |  |  |
|  |  |  |  |  |  |  |
| Number of episodes of severe diarrhea^8^ |  |  |  |  |  |  |
| Below the median | 329 | 31.00 | 1 | 0.26 |  |  |
| Above the median | 113 | 37.17 | 1.29 (0.82-2.02) |  |  |  |
|  |  |  |  |  |  |  |
| Number of hospital episodes^8^ |  |  |  |  |  |  |
| Below the median | 427 | 32.32 | 1 | 0.59 |  |  |
| Above the median | 15 | 40.00 | 1.34 (0.44-3.80) |  |  |  |

Abbreviations: OR; Odds Ratio, CI; Confidence Interval, ORS; Oral rehydration salts.

^1^Minimally-adjusted models are adjusted for age at baseline and child sex. ^2^Hygiene score was calculated as the sum of the scores for the following variables: “Wash hands after helping child defecate”; “Wash hands before preparing food”; “Wash hands after using toilet”; and “Uses toilet paper”. Each variable was scored as follows: 1 = never, 2 = rarely, 3 = sometimes, 4 = always. ^3^Asset scores were the sum of two sub-scores: asset score 1, which was the sum of the following possessions: iron, chair/bench, sofa, table, computer, fridge, motorcycle, and bank account; asset score 2, which was the sum of the following possessions: electric fan, television, mattress, mobile phone. Asset score could range from 4 to 16. ^4^As defined by the Household Food Insecurity Asset Scale (HFIAS). ^5^Wasted defined as weight-for-length z-score <-2 standard deviations. ^6^Stunted defined as length-for-age z-score <-2 standard deviations. ^7^Underweight defined as weight-for-age z-score <-2. ^8^Morbidity variable definitions can be found Supporting Information: Table 2

**Supplementary Table 5:** Prevalence of Iron Deficiency Anemia (IDA) among children enrolled in the biochemistry subgroup of the Zinc in Powders trial (ZiPT) using updated hemoglobin cutoffs for anemia (hemoglobin <10.5 d/dL)

| Biochemistry Subgroup: Standard MNP | Prevalence of IDA^1^ |
| --- | --- |
| Baseline (N=58) | 46.55% (27) |
| 24 weeks (N=50) | 16.00% (8) |

^1^IDA defined as the presence of both anemia (hemoglobin < 10.5 g/dL) and low serum ferritin (ferritin < 12/ugL). Ferritin values were adjusted for inflammation (C-reactive protein (CRP) and alpha(1)-Acid glycoprotein (AGP)) using the BRINDA regression correction approach.

Reference:

Islam, M. M., Black, R. E., Krebs, N. F., Westcott, J., Long, J. M., Islam, K. M., Peerson, J. M., Sthity, R. A., Khandaker, A. M., Hasan, M., El Arifeen, S., Ahmed, T., King, J. C., & McDonald, C. M. (2022, Dec 15). Effects of Different Doses, Forms, and Frequencies of Zinc Supplementation on Biomarkers of Iron and Zinc Status among Young Children in Dhaka, Bangladesh. *Nutrients, 14*(24). <https://doi.org/10.3390/nu14245334>

**Supplementary Figure 1**: Density plot of hemoglobin concentrations at 24 weeks

**
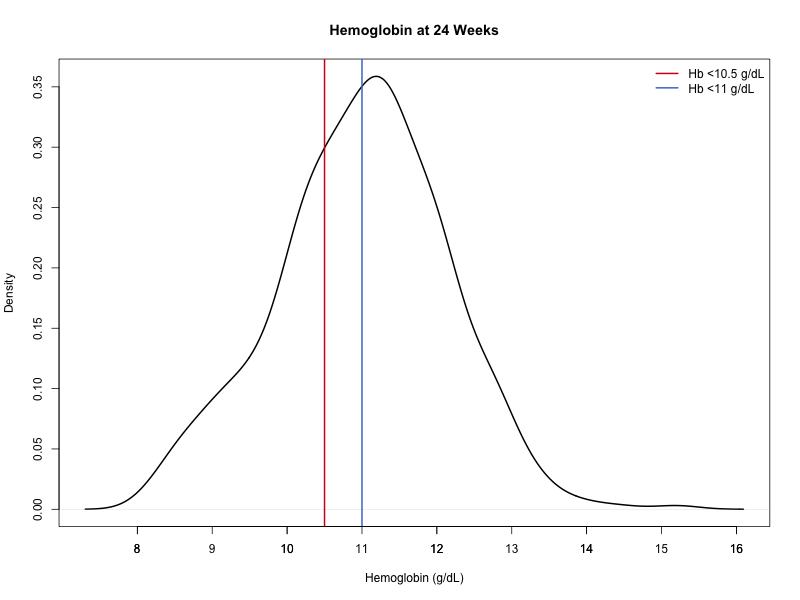
**

**Supplementary Figure 2**: Heatmap of predictors of anemia (hemoglobin <11 g/dL) among children enrolled in the standard MNP daily group

^1^Variables are only shown if they are associated (p<0.1) with anemia at endline (24 weeks) in either minimally-adjusted bivariate or multivariate logistic regression models. ^2^Variables described as lower assume the value of the lower (binary) or lowest variable category. Variables described as higher assume the value of the higher (binary) or highest variable category.


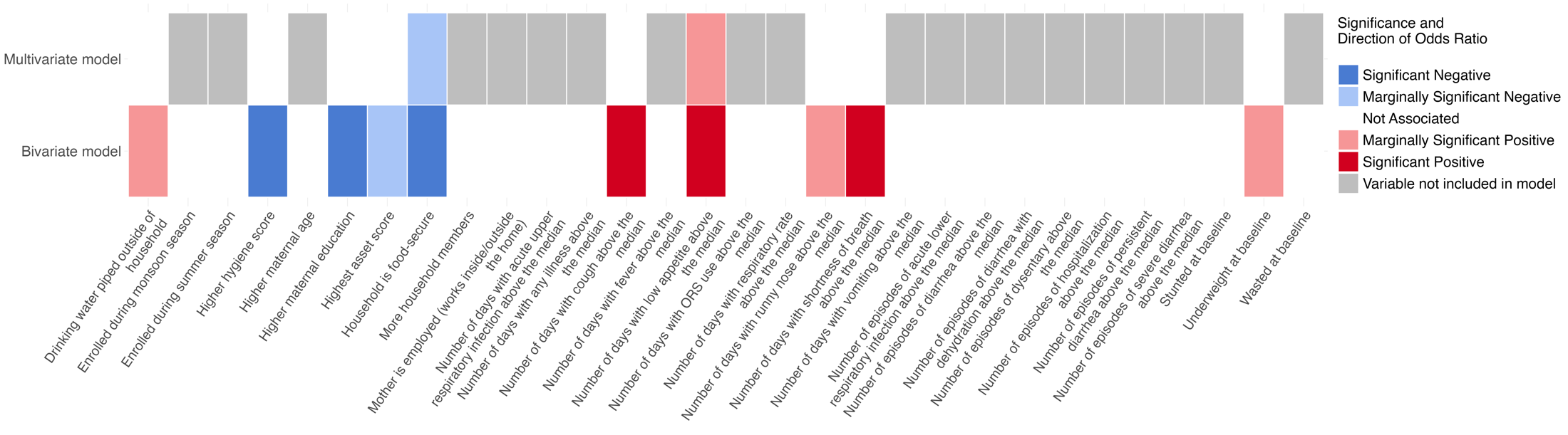


**Supplementary Figure 3**: Heatmap of predictors of anemia (hemoglobin <10.5 g/dL) among children enrolled in the standard micronutrient powder (MNP) daily group including baseline anemia status as a predictor


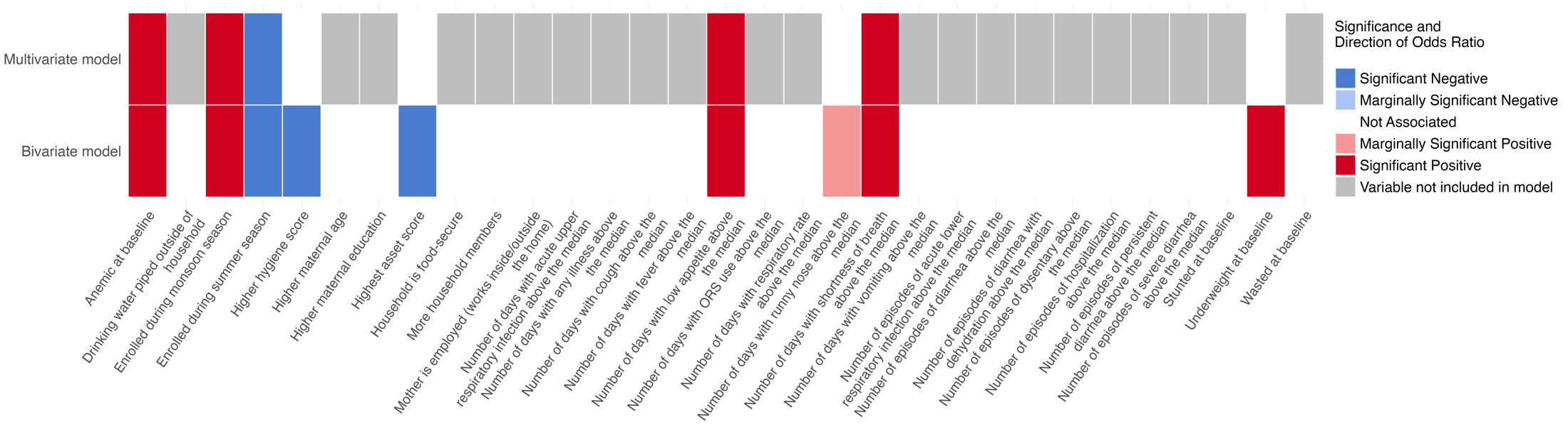


^1^Variables are only shown if they are associated (p<0.1) with anemia at endline (24 weeks) in either minimally-adjusted bivariate or multivariate logistic regression models. ^2^Variables described as lower assume the value of the lower (binary) or lowest variable category. Variables described as higher assume the value of the higher (binary) or highest variable category.
